# Origin of Pathogens of Grapevine Crown Gall Disease in Hokkaido in Japan as Characterized by Molecular Epidemiology of *Allorhizobium vitis* Strains

**DOI:** 10.3390/life11111265

**Published:** 2021-11-19

**Authors:** Akira Kawaguchi, Teruo Sone, Sunao Ochi, Yosuke Matsushita, Yoshiteru Noutoshi, Mizuho Nita

**Affiliations:** 1 Western Region Agricultural Research Center (WARC) (Kinki, Chugoku, and Shikoku Regions), National Agriculture and Food Research Organization (NARO), 6-12-1 Nishifukatsu-cho, Fukuyama, Hiroshima 721-8514, Japan; 2 Research Faculty of Agriculture, Hokkaido University, Kita 9 Nishi 9, Kita-ku, Sapporo 060-8589, Japan; sonet@chem.agr.hokudai.ac.jp; 3 Institute of Plant Protection, National Agriculture and Food Research Organization (NIPP), 2-1-18 Kannondai, Tsukuba, Ibaraki 721-8514, Japan; sunaochi@affrc.go.jp (S.O.); yosuken@affrc.go.jp (Y.M.); 4 Graduate School of Environmental and Life Science, Okayama University, 1-1-1 Tsushima-naka, Kita-ku, Okayama 700-8530, Japan; noutoshi@okayama-u.ac.jp; 5 Alson H. Smith, Jr. Agricultural Research and Extension Center, School of Plant and Environmental Sciences, Virginia Polytechnic Institute and State University, Winchester, VA 22602, USA; nita@vt.edu

**Keywords:** *Rhizobium vitis*, multi-locus sequence analysis, grapevine crown gall, vineyard, epidemic

## Abstract

Crown gall is a globally distributed and economically important disease of grapevine and other important crop plants. The causal agent of grapevine crown gall is tumorigenic *Allorhizobium vitis* (Ti) strains that harbor a tumor-inducing plasmid (pTi). The epidemic of grapevine crown gall has not been widely elucidated. In this study, we investigated the genetic diversity of 89 strains of Ti and nonpathogenic *A. vitis* to clarify their molecular epidemiology. Multi-locus sequence analysis (MLSA) of the partial nucleotide sequences of *pyrG, recA*, and *rpoD* was performed for molecular typing of *A. vitis* strains isolated from grapevines with crown gall symptoms grown in 30 different vineyards, five different countries, mainly in Japan, and seven genomic groups A to F were obtained. The results of MLSA and logistic regression indicated that the population of genetic group A was significantly related to a range of prefectures and that the epidemic of group A strains originated mainly in Hokkaido in Japan through soil infection. Moreover, group E strains could have been transported by infected nursery stocks. In conclusion, this study indicates that both soil infection and transporting of infected nursery stocks are working as infection source in Hokkaido.

## 1. Introduction

Grapevine (*Vitis vinifera* L.) crown gall is caused mainly by tumorigenic *Allorhizobium vitis* (syn. *Rhizobium vitis* (Ti), *Agrobacterium* *vitis* (Ti), *A. tumefaciens* biovar 3, where “Ti” means “tumorigenic” or “tumor-inducing”). In this paper, we follow the nomenclature for *Allorhizobium* species adopted by Mousavi et al. [1] to avoid confusion. This pathogen enters the grapevine through wounds due to a variety of causes, such as cold injury, mechanical damage, and grafting [2]. *A. vitis* (Ti) causes crown gall by transferring the T-DNA region of the tumor-inducing bacterial plasmid (Ti-plasmid) to the host cell, which subsequently integrates into the plant host genome [3,4,5]. The inserted T-DNA contains genes for biosynthesis of plant growth hormones [6,7]. Subsequent expression of T-DNA genes results in the overproduction of auxins and cytokinins, which eventually leads to abnormal gall formation in the host plant. DNA genes then produce tumor-specific compounds called opines, which serve as nutrients for *A. vitis* [7]. Invasion of vascular tissue by galls can result in vine death [8,9].

There is no effective method to control grapevine crown gall that can be used in commercial fields so far. Previously, we reported that a nonpathogenic *A. vitis* strain, VAR03-1, which was isolated from grapevine nursery stock in Japan, inhibited tumor formation in grapevine, rose, tomato, sunflower, and apple [10,11,12,13,14,15]. Moreover, we identified a nonpathogenic strain ARK-1 as a new antagonistic strain [16,17,18,19,20,21,22]. ARK-1 does not have the Ti-plasmid, so ARK-1 neither carries nor causes disease symptoms [16]. It provided better control against grapevine crown gall than VAR03-1 in field trials, and pretreatment of grapevine roots with ARK-1 cell suspension before planting in Ti-contaminated soil effectively suppressed gall formation in roots [16,17,20].

To apply biological control agents ARK-1 and/or VAR03-1 for management of crown gall in commercial vineyards effectively and efficiently, it is essential to know the epidemics of this disease. Crown gall infection takes place not only in vineyards, but also in nurseries [7]. With nursery production, symptoms develop at the site of wounds made by disbudding, at the base of rooted cuttings, and at grafts; however, in many cases, the infected plants remain symptomless until frost or other physical damage initiates the disease [7,23]. Therefore, Ti strains are often transmitted through the vegetative propagation of infected asymptomatic grapevines. When mother vines at a nursery are infected, the pathogen can be spread very quickly through a production and dissemination of nursery stocks. Recently, grapevine crown gall has often occurred in many vineyards in Japan, but it is unclear whether the major infection route of recent outbreaks is soil-borne or transmission of nursery stocks or both.

Thus, the objectives of this study are to classify the genetic diversity of 89 strains of *A. vitis* obtained from diseased grapevines by multi-locus sequence analysis (MLSA) of the partial nucleotide sequences of housekeeping genes and to clarify the molecular epidemiology of *A. vitis* strains collected in various locations in Japan and other four countries.

## 2. Materials and Methods

### 2.1. Multi-Locus Sequence Analysis (MLSA)

The *A. vitis* including Ti and nonpathogenic strains used in this study are listed in Table 1 and Supplementary Table S1. The sources of the strains and their relevant characteristics have been described in previous papers [16,22,24,25,26,27]. The 89 *A. vitis* strains were isolated from 19 varieties of grapevine cultivars (including three unknown cultivars), 30 different vineyard locations, 13 different prefectures or states, five different countries (Japan, USA, Iran, Australia, and Greece), and different decades (before 2000, 2000 to 2009, 2010 to 2019, and after 2020) (Table 1). The multiplex polymerase chain reaction (PCR) was performed using a mixture of two primer sets Ab3-F3 ⁄ Ab3-R4 and VCF3 ⁄ VCR3 to identify Ti and non-pathogenic strains of *A. vitis* according to the procedure of previous reports [11,12,28]. Our previous reports [24,28] have shown that the MLSA approach using three housekeeping genes *pyrG* (CTP synthetase), *recA* (recombinase A), and *rpoD* (RNA polymerase, sigma 70 factor) was useful to reveal the genetic diversity of *A. vitis* strains. In this study, we followed the experimental methods described in previous reports [24,29]. PCR amplifications of *pyrG*, *recA*, and *rpoD* genes were performed using primers ApyrF1 and ApyrR4, recAF1 and recAR2, and ArpoF1 and ArpoR2, respectively, as reported by Kawaguchi et al. [29] and Kawaguchi [24]. The PCR reactions were conducted using LifeECO ver3.0 (Nippon Genetics Co., Ltd., Tokyo, Japan). The partial nucleotide sequences of *pyrG* (849 bp)*, recA* (465 bp), and *rpoD* (733 bp) of the 79 strains of Ti and ten nonpathogenic strains were directly determined from the PCR products using ApyrF1 and ApyrR4, recAF2 and recAR2, and ArpoF3 and ArpoR2 as sequencing primers, respectively (Kawaguchi et al. 2008b; Kawaguchi 2011). The data for the *pyrG*, *recA*, and *rpoD* sequences of 44 strains (accession numbers AB272143 to AB608986) were obtained in previous studies [24,29] and downloaded from the DDBJ database (http://getentry.ddbj.nig.ac.jp) (accessed on 18 October 2021), and those of 45 strains (accession number from LC629040 to LC635338) were newly obtained by sequence analysis using Big Dye Terminator v.3.1 cycle sequencing kit (Applied Biosystems, Foster City, CA, USA) in this study (Supplementary Table S1). The concatenated sequence data for *pyrG*, *recA*, and *rpoD* were aligned with MEGA X software (http://www.megasoftware.net/) (accessed on 18 October 2021) [30]. Maximum likelihood (ML), neighbor-joining (NJ), and minimum-evolution (ME) trees were constructed, and the strength of the internal branches from the resulting tree was tested by bootstrap analysis using 1000 replications.

### 2.2. Logistic Regression

The *A. vitis* strains were adequate to perform the statistical analysis due to the large sample size (89 strains) and because they were isolated from various cultivars, locations, countries, and isolation-year histories. In this study, we followed the experimental methods described in a previous report [30]. The *A. vitis* strains were binary-coded as either 1 (belonging to one specific genetic group) or 0 (belonging to the other genetic groups). The parameters, which were years, cultivars, vineyard locations, prefectures/states, or countries, and when/where *A. vitis* strains were isolated, were also coded using a binary scale (1 or 0) based on categorical numbers as well as genetic group (Supplementary Table S1).

The logistic regression model was defined as:

ln{P/(1 − P)} = α + β_1_·x_1_ + β_2_·x_2_ + … + β_n_·x_n_,, (1)

where P is the proportion of *A. vitis* strains belonging to one specific genetic group, α is the y-intercept, and β_n_ is the coefficient associated with predictor variable x_n_. According to previously described procedures [16,31], the R (ver. 3.6.1, R Development Core Team) package “glm” was used for calculation of logistic regression coefficients. The link function was the logit. The stepwise selection of the explanatory variables was based on the value of Akaike’s information criterion (AIC).

### 2.3. Odds Ratio

The relationship between the genetic group A and factors was calculated as an odds ratio (OR). An OR was defined as:

OR = {P_a_/(1 − P_a_)]/[P_o_/(1/P_o_)}, (2)

where P_a_ is the proportion of genetic group A strains isolated in Hokkaido and P_o_ is the proportion of genetic group A strains isolated in other prefectures/states (except in Hokkaido). An OR is a measure of association between an exposure and an outcome. The OR represents the odds that an outcome will occur given a particular exposure, compared with the odds of the outcome occurring in the absence of that exposure. In the present study, a high OR indicates a high probability of appearance of genetic group A strains in Hokkaido, and a low OR indicates a low probability of them appearing.

## 3. Results

### 3.1. Multi-Locus Sequence Analysis (MLSA)

**Figure 1:**
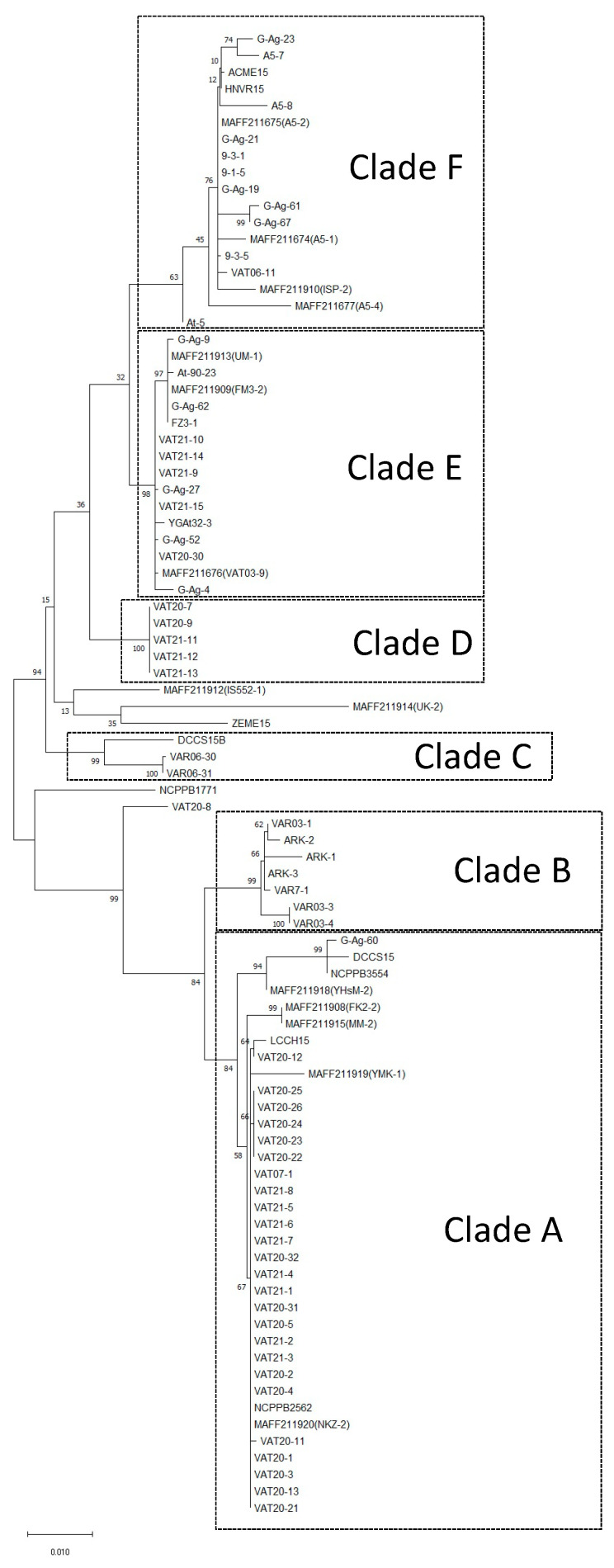
Phylogenetic tree of *Allorhizobium* *vitis* strains based on the maximum likelihood (ML) method using concatenated sequence data for *pyrG*, *recA*, and *rpoD*. Bootstrap values from 1000 samplings are indicated. The bar represents a phylogenetic distance of 1%.

In the phylogenetic tree constructed by the ML method using the combined sequence data of three housekeeping genes (*pyrG*, *recA*, and *rpoD*), the 89 *A. vitis* strains separated into six clades (A to F) (Figure 1, Table 1). The 79 Ti strains used in this study comprised four genetic groups, and 35, 5, 18, and 16 strains belonged to genetic groups A, D, E, and F, respectively (Figure 1, Table 1). The ten nonpathogenic strains separated into two genetic groups, with seven and three strains belonging to genetic groups B and C, respectively (Figure 1, Table 1). The topology of the phylogenic tree based on the ML method perfectly coincided with that based on the NJ and ME methods, indicating that the divisions for the six clades in the phylogenic tree were valid (Figure 1, Figures S1 and S2). Five Ti strains (VAT20-8, MAFF211912, MAFF211914, ZEME15, and NCPPB1771) neither belonged to clade A to F and nor formed a clade based on ML, NJ, and ME methods (Figure 1, Figures S1 and S2, Table 1).

### 3.2. Logistic Regression

Genetic groups A, E, or F have more *A. vitis* strains (35, 18, and 16, respectively) than those B, C, and D (Figure 1, Table 1). The relationship with the records of isolation history, which were years, cultivars, vineyard locations, prefectures/states, and countries of the strains belonging to A, E, or F were investigated. In genetic group A, a logistic regression with a stepwise selection method based on AIC was conducted; two factors “Yoichi” (in the category “location of vineyards”) and “Hokkaido” (in “prefecture/state”) were selected as variables, but a variable of “Hokkaido” was only significantly correlated with the objective variable (*p* = 4.5 × 10^−4^, Table 2). In genetic groups E and F, no factor was significantly selected as a variable by a logistic regression with a stepwise selection method based on AIC (data not shown).

### 3.3. Odds Ratio

The odds ratio, which was obtained from the logistic regression used to predict the proportion of genetic group A strains isolated from grapevines in Hokkaido, was 10.52 (95% confidence interval = 3.68 to 30.68, *p* = 0.048). This result indicated that there was a significantly high probability of appearance of genetic group A strains in Hokkaido.

## 4. Discussion

In our previous report (Kawaguchi 2011), 35 Ti strains were separated into five (previous clades A to E) groups and six nonpathogenic strains into two (previous clades F and G) groups. However, previously determined clades D and E had two Ti strains MAFF211912 (IS552-1) and MAFF211914 (UK-2), respectively [24]. In this study, these two Ti strains were not grouped as clades because different nodes were obtained from ML, NJ, and ME phylogenetic trees and low bootstrap values (<50%, Figure 1, , Figures S1 and S2, Table 1). Moreover, a new genetic group D, which had five Ti strains isolated from *V. vinifera* cv. Kerner and cv. Zweigeltrebein in Urausu, Hokkaido, Japan, was revealed (Figure 1, Table 1). All Ti strains belonging to group D were isolated from two different cultivars in the same vineyard in 2020, but it was unclear where these strains came from because logistic regression could not be carried out using only five strains.

The genetic groups B and C in this study coincided with previous groups F and G, respectively (Figure 1) [24]. The group B strains, which were nonpathogenic strains including VAR03-1 and ARK-1, were antagonistic against Ti strains [10,11,12,13,14,15,16,17,18,19,20,21,22,29,32], but the nonpathogenic strains belonging to group C were not. This result suggests that the housekeeping genes *pyrG*, *recA*, and *rpoD* in *A. vitis* strains, which are antagonistic to grapevine crown gall, are genetically dissimilar from those of non-antagonistic strains.

The genetic groups A, E, and F in this study coincided with previous groups C, A, and B, respectively (Figure 1) [24]. Groups A, E, and F have many Ti strains derived from various districts of Japan (Figure 1, Table 1). Group E has 18 Ti strains isolated in Japan and Virginia, USA. Japanese strains in group E were isolated from various districts in nine different prefectures, indicating that Ti strains of group E could be widely distributed around Japan. It is still unclear that these strains originally lived in the soil in each district or were moved by circulation of infected nursery stocks. Although no factors were significantly selected as variables by a logistic regression, these strains were isolated in two different countries—Japan and the USA (Table 1). In Japan, there are large nursery production vineyards in several prefectures including group E, and nursery stocks of various grapevine cultivars are distributed from some prefectures to all areas of Japan. These results indicate that strains in group E could have been moved by circulation of infected nursery stocks.

Group F has 16 Ti strains isolated in Japan alone. These strains were also isolated from various districts in six different prefectures, indicating that Ti strains in group F could be as widely distributed around Japan as group E. Many strains in group F (12/16) were isolated from *V*. *labrusca* × *V*. *vinifera* cv. Kyoho, which is grown in all over Japan because it is very common as a table grape in Japan, and some strains in group E were also isolated from cv. Kyoho (Table 1). However, various cultivators were not significantly selected as variables by a logistic regression. The small sample size (n = 16) might be insufficient for logistic regression. In our future studies, more strains are needed to certify the relationship between genetic groups and cultivar varieties.

Group A has 35 Ti strains (including with type strain NCPPB3554^T^) isolated in Japan, USA, Australia, and Greece, indicating that Ti strains of group A could be widely distributed around many countries. In the results of the stepwise regression analysis focusing on the group A strains, only the variable “Hokkaido” was selected as a significantly correlated parameter explaining the group A population. According to the OR results, there is also a significantly high probability of the appearance of group A strains in Hokkaido. These results indicate that group A population is significantly related to Hokkaido. Growers usually buy nursery stocks from grapevine nursery production vineyards in prefectures other than Hokkaido because nursery stocks are rarely produced in Hokkaido. In this study, however, group A strains were never isolated in these three nursery production prefectures (Table 1). Thus, these findings indicate that the group A strains would have been already disseminated in Hokkaido before 2000 and that crown gall could have been mainly caused by group A strains not via circulation of infected nursery stocks but by soil infection at each vineyard after 2000.

In this study, some Ti strains collected in various locations in other four countries except Japan, some Ti strains (including the type-strain NCPPB3554^T^) isolated from USA, Australia, and Greece belonged to genetic group A, indicating that group A might be one of the major genetic groups around the world. On the other hand, two Ti strains ACME15 and HNVR15 collected in USA were belonging to genetic group F, which had also 17 Ti strains collected in Japan. To verify whether the group F is a specific group be distributed between Japan and Virginia, an additional study of investigation of more varieties of A. vitis Ti strains collected in various countries is needed.

Five Ti strains (VAT20-8, MAFF211912, MAFF211914, ZEME15, and NCPPB1771) did not belong to genetic groups A to F using sequences data of *pyrG*, *recA*, and *rpoD* (Figure 1, Table 1). These five strains might be formed a clade by other housekeeping genes instead of *pyrG*, *recA*, and *rpoD*. Moreover, the authors should try with other genes such as additional housekeeping genes RNA genes for further confirmation of MLSA.

In general, freeze injuries provide sites for initiating crown gall [7,33]. Severe winter weather, as well as recent trends in extreme temperature fluctuations during late winter and early spring, tend to damage grapevine trunks, which allows the entry of *A. vitis* (Ti) [7]. Hokkaido is a cold region, and vines are also exposed to freeze injuries. Grapevine crown gall has been increasing in Hokkaido since 1990 [34,35]. Recently, grapevines in many vineyards were damaged by crown gall disease in Hokkaido, and many Ti strains belonging into three genetic groups (A, D, and E) have been isolated after 2020 (Figure 1, Table 1). Our results indicate that the occurrence of crown gall in Hokkaido could be due to both soil infection caused by group A strains and entry of infected nursery stocks by group E strains. If biological control agents ARK-1 and/or VAR03-1 are applied to control crown gall in Hokkaido, the roots of pathogen-free nursery stocks should be treated with ARK-1 and/or VAR03-1 (e.g., dipping into cell suspension of antagonists) before planting to prevent soil infection. To produce pathogen-free nursery stocks, moreover, antagonistic strains should be applied in nurseries in prefectures other than Hokkaido.

The genetic diversity of *A. vitis* (Ti) isolated in some countries has previously been reported [22,36,37]. The results from cluster analysis based on repetitive sequence-based (rep)-PCR and inter-simple sequence-repeat (ISSR)-PCR data concurrently showed a potential genomic diversity that separates the Virginia strains from the Japanese strains using a total of 12 strains [22]. However, results from MLSA showed some Virginia strains and Japanese strains formed the same cluster (strain LCCH15 belonging to group A, DCCS15B to group B, ACME15 and HNVR15 to group E) (Figure 1, Table 1). Kuzmanović et al. [36] reported that genetic varieties of *A. vitis* (Ti) of 29 strains isolated in European countries and the USA were analyzed by random amplified polymorphic DNA (RAPD) PCR, and sequence analysis of housekeeping genes of *dnaK*, *gyrB,* and *recA*. RAPD divided them into 17 groups, but a phylogenetic tree based on *recA* gene divided them into four clades [36]. It seems that PCR-based analysis tends to divide into more groups than partial sequence analysis. However, concurrently amplifying many PCR fragments of different lengths is sometimes unreliable and strains should be compared among using results of the band patterns obtained from concurrent PCR reactions and in the same gel. Thus, genetic performing diversity analysis using PCR and gel electrophoresis for many strains isolated in several countries is sometimes difficult. On the other hand, although the results would not reflect the whole genome information, partial sequence analysis is robust and could be used on the deposited sequence data in the public DNA databases (e.g., DDBJ/EMBL/GenBank), even strains conserved in each country. Recently, whole genome sequences of three *A. vitis* strains used in this study (MAFF211676 (former name VAT03-9), VAR03-1, and VAR06-30) are already available [38,39,40]. If complete genome sequence data of *A. vitis* strains obtained by a next generation sequencing system are accumulated, assessment of genetic diversity using them could become easy. By knowing the diversity of *A. vitis*, we can now select representative strains to determine the effectiveness of disease control strategies. For example, the effectiveness of biological controls can be determined for a diverse group of strains that are representative of the different genetic groups. Our group has already reported that the nonpathogenic *A. vitis* strain ARK-1 inhibited formation of galls caused by representative Ti strains in group A, E, and F in this study, which coincided with previous groups C, A, and B, respectively [15,18,22]. We plan to test group D and non-clustered Ti strains in biological control experiments.

## 5. Conclusions

The results of MLSA of the partial nucleotide sequences of *pyrG, recA*, and *rpoD* and of logistic regression analyses indicated that the population of genetic group A was significantly related to a range of prefectures and that the epidemic of group A strains could have originated in the Hokkaido region mainly through soil infection. Moreover, group E strains could have been moved by circulation of infected nursery stocks. In conclusion, this study indicated that both soil infection and transporting of infected nursery stock were working as infection sources in Hokkaido. This study will be applicable to future studies of the molecular epidemiology of grapevine crown gall occurring in several countries.

## Figures and Tables

**Table 1:** List of *Allorhizobium* *vitis* strains analyzed.

Strains (Former Name)	Ti or N ^a^	Cultivar ^b^	Location of Vineyard	Prefcture/State	Country	Isolated Year	Genetic Group ^c^
MAFF663001 (G-Ag-27)	Ti	Kyoho	Matsumoto	Nagano	Japan	Before 2000	E
MAFF212292 (YGAt32-3)	Ti	Garnet A	Yamanashi	Yamanashi	Japan	Before 2000	E
MAFF663017 (G-Ag-4)	Ti	Kyoho	Shimane	Shimane	Japan	Before 2000	E
MAFF663004 (G-Ag-9)	Ti	Kyoho	Yokota	Shimane	Japan	Before 2000	E
MAFF211676 (VAT03-9)	Ti	Seto Giants	Asaguchi	Okayama	Japan	2000 to 2009	E
MAFF211944 (G-Ag-62)	Ti	Kyoho	Sagae	Yamagata	Japan	Before 2000	E
MAFF211889 (G-Ag-52)	Ti	Kyoho	Hanamaki	Iwate	Japan	Before 2000	E
At-90-23	Ti	Kyoho	Shimane	Shimane	Japan	Before 2000	E
FZ-3-1	Ti	Zweigeltrebe	Furano	Hokkaido	Japan	2000 to 2009	E
VAT20-30	Ti	Pinot Noir	Chitose	Hokkaido	Japan	After 2020	E
VAT21-9	Ti	Zweigeltrebe	Yoichi	Hokkaido	Japan	After 2020	E
VAT21-10	Ti	Kerner	Yoichi	Hokkaido	Japan	After 2020	E
VAT21-14	Ti	Cambell Early	Yoichi	Hokkaido	Japan	After 2020	E
VAT21-15	Ti	Cambell Early	Yoichi	Hokkaido	Japan	After 2020	E
ACME15	Ti	Merlot	Winchester	Virginia	USA	2010 to 2019	E
HNVR15	Ti	Viognier	Gordonsville	Virginia	USA	2010 to 2019	E
MAFF211909 (FM-3-2)	Ti	Müller-Thurgau	Furano	Hokkaido	Japan	2000 to 2009	E
MAFF211913 (UM-1)	Ti	Müller-Thurgau	Urausu	Hokkaido	Japan	2000 to 2009	E
MAFF211301 (At-5)	Ti	Kyoho	Shimane	Shimane	Japan	Before 2000	F
MAFF211674 (A5-1)	Ti	Kyoho	Yokote	Akita	Japan	2000 to 2009	F
MAFF211675 (A5-2)	Ti	Kyoho	Yokote	Akita	Japan	2000 to 2009	F
MAFF211677 (A5-4)	Ti	Kyoho	Yokote	Akita	Japan	2000 to 2009	F
A5-7	Ti	Kyoho	Yokote	Akita	Japan	2000 to 2009	F
MAFF211302 (A5-8)	Ti	Kyoho	Yokote	Akita	Japan	2000 to 2009	F
VAT06-11	Ti	Aurora Black	Ukan	Okayama	Japan	2000 to 2009	F
MAFF663006 (G-Ag-19)	Ti	Rizamat	Shiojiri	Nagano	Japan	Before 2000	F
MAFF663007 (G-Ag-21)	Ti	Kyoho	Shiojiri	Nagano	Japan	Before 2000	F
MAFF663008 (G-Ag-23)	Ti	Kyoho	Shiojiri	Nagano	Japan	Before 2000	F
9-1-5	Ti	Kyoho	Hanamaki	Akita	Japan	2000 to 2009	F
9-3-1	Ti	Kyoho	Hanamaki	Akita	Japan	2000 to 2009	F
9-3-5	Ti	Kyoho	Hanamaki	Akita	Japan	2000 to 2009	F
MAFF211943 (G-Ag-61)	Ti	Beniizu	Sannohe	Aomori	Japan	Before 2000	F
MAFF211949 (G-Ag-67)	Ti	Kyoho	Yokote	Akita	Japan	Before 2000	F
MAFF211910 (ISP-2)	Ti	Pinot Noir	Ikeda	Hokkaido	Japan	2000 to 2009	F
MAFF212306 (VAR03-1)	N	Seto Giants	Okayama	Okayama	Japan	2000 to 2009	B
ARK-1	N	Pione	Okayama	Okayama	Japan	2000 to 2009	B
MAFF212307 (VAR03-3)	N	Seto Giants	Okayama	Okayama	Japan	2000 to 2009	B
MAFF212308 (VAR03-4)	N	Seto Giants	Okayama	Okayama	Japan	2000 to 2009	B
ARK-2	N	Pione	Okayama	Okayama	Japan	2000 to 2009	B
ARK-3	N	Pione	Okayama	Okayama	Japan	2000 to 2009	B
MAFF212313 (VAR7-1)	N	Seto Giants	Okayama	Okayama	Japan	2000 to 2009	B
VAR06-30	N	Aurora Black	Ukan	Okayama	Japan	2000 to 2009	C
VAR06-31	N	Aurora Black	Ukan	Okayama	Japan	2000 to 2009	C
DCCS15B	N	Cabernet Sauvignon	Etlan	Virginia	USA	2010 to 2019	C
NCPPB3554^T^	Ti	Unknown	Unknown	Unknown	Australia	Before 2000	A
DCCS15	Ti	Cabernet Sauvignon	Etlan	Virginia	USA	2010 to 2019	A
MAFF211942 (G-Ag-60)	Ti	Cambell Early	Nanbu	Aomori	Japan	2000 to 2009	A
MAFF211918 (YHsM-2)	Ti	Müller-Thurgau	Yoichi	Hokkaido	Japan	2000 to 2009	A
VAT07-1	Ti	Aurora Black	Asaguchi	Okayama	Japan	2000 to 2009	A
NCPPB2562	Ti	Unknown	Unknown	Unknown	Greece	Before 2000	A
MAFF211919 (YMK-1)	Ti	Kerner	Yoichi	Hokkaido	Japan	2000 to 2009	A
MAFF211920 (NKZ-2)	Ti	Zweigeltrebe	Niki	Hokkaido	Japan	2000 to 2009	A
MAFF211908 (FK-2-2)	Ti	Kerner	Furano	Hokkaido	Japan	2000 to 2009	A
MAFF211915 (MM-2)	Ti	Müller-Thurgau	Mikasa	Hokkaido	Japan	2000 to 2009	A
LCCH15	Ti	Chardonnay	Charlottesville	Virginia	USA	2010 to 2019	A
VAT20-1	Ti	Zweigeltrebe	Urausu	Hokkaido	Japan	After 2020	A
VAT20-2	Ti	Zweigeltrebe	Urausu	Hokkaido	Japan	After 2020	A
VAT20-3	Ti	Kerner	Urausu	Hokkaido	Japan	After 2020	A
VAT21-4	Ti	Kerner	Urausu	Hokkaido	Japan	After 2020	A
VAT21-5	Ti	Kerner	Urausu	Hokkaido	Japan	After 2020	A
VAT20-11	Ti	Zweigeltrebe	Yoichi	Hokkaido	Japan	After 2020	A
VAT20-12	Ti	Kerner	Yoichi	Hokkaido	Japan	After 2020	A
VAT20-13	Ti	Zweigeltrebe	Yoichi	Hokkaido	Japan	After 2020	A
VAT20-21	Ti	Kerner	Niseko	Hokkaido	Japan	After 2020	A
VAT20-22	Ti	Kerner	Niseko	Hokkaido	Japan	After 2020	A
VAT20-23	Ti	Kerner	Niseko	Hokkaido	Japan	After 2020	A
VAT20-24	Ti	Kerner	Niseko	Hokkaido	Japan	After 2020	A
VAT20-25	Ti	Kerner	Niseko	Hokkaido	Japan	After 2020	A
VAT20-26	Ti	Kerner	Niseko	Hokkaido	Japan	After 2020	A
VAT20-31	Ti	Pinot Noir	Chitose	Hokkaido	Japan	After 2020	A
VAT20-32	Ti	Pinot Noir	Chitose	Hokkaido	Japan	After 2020	A
VAT21-1	Ti	Zweigeltrebe	Yoichi	Hokkaido	Japan	After 2020	A
VAT21-2	Ti	Zweigeltrebe	Yoichi	Hokkaido	Japan	After 2020	A
VAT21-3	Ti	Zweigeltrebe	Yoichi	Hokkaido	Japan	After 2020	A
VAT21-4	Ti	Zweigeltrebe	Yoichi	Hokkaido	Japan	After 2020	A
VAT21-5	Ti	Zweigeltrebe	Yoichi	Hokkaido	Japan	After 2020	A
VAT21-6	Ti	Zweigeltrebe	Yoichi	Hokkaido	Japan	After 2020	A
VAT21-7	Ti	Zweigeltrebe	Yoichi	Hokkaido	Japan	After 2020	A
VAT21-8	Ti	Zweigeltrebe	Yoichi	Hokkaido	Japan	After 2020	A
VAT20-7	Ti	Zweigeltrebe	Urausu	Hokkaido	Japan	After 2020	D
VAT20-9	Ti	Zweigeltrebe	Urausu	Hokkaido	Japan	After 2020	D
VAT21-11	Ti	Zweigeltrebe	Urausu	Hokkaido	Japan	After 2020	D
VAT21-12	Ti	Zweigeltrebe	Urausu	Hokkaido	Japan	After 2020	D
VAT21-13	Ti	Kerner	Urausu	Hokkaido	Japan	After 2020	D
VAT20-8	Ti	Zweigeltrebe	Urausu	Hokkaido	Japan	After 2020	nc
ZEME15	Ti	Merlot	Hamilton	Virginia	USA	2010 to 2019	nc
MAFF211912 (IS552-1)	Ti	Pinot Noir	Ikeda	Hokkaido	Japan	2000 to 2009	nc
MAFF211914 (UK-2)	Ti	Kerner	Urausu	Hokkaido	Japan	2000 to 2009	nc
NCPPB1771	Ti	Unknown	Unknown	Unknown	Iran	Before 2000	nc

^a^ Ti: Tumorigenic. N: Nonpathogenic. ^b^ indicates cultivar name of grapevine; *Vitis labrusca* × *V. vinifera* cv. Kyoho; *V. vinifera* cv. Garnet A; *V. vinifera* cv. Seto Giants; *V. labrusca* × *V. vinifera* cv. Cambell Early; *V. vinifera* × *V. labrusca* cv. Pione; *V. vinifera* cv. Kerner; *V. vinifera* cv. Zweigeltrebe; *V. vinifera* cv. Müller-Thurgau; *V. vinifera* cv. Rizamat; *V. vinifera* × *V. labrusca* cv. Seibel5279; *Vitis* sp. cv. Aurora Black; *Vitis* sp. cv. Beniizu; *V. vinifera* cv. Merlot; *V. vinifera* cv. Cabernet Sauvignon; *V. vinifera* cv. Chardonnay; *V. vinifera* cv. Viognier; *V. vinifera* cv. Pinot Noir. ^c^ nc: not clustered.

**Table 2:** Parameter estimates ^a^ for the logistic regression model used to predict the proportion of *A. vitis* strains in genetic group A.

Variable		Parameter Estimate	Standard Error	*z* Value	*p* Value ^b^
Category	Factor				
Location of vineyard	Yoichi	0.971	0.683	1.422	0.155
Prefecture/state	Hokkaido	2.027	0.577	3.512	4.5 × 10^−4^
*y*-Intercept		−1.819	0.440	−4.133	3.6 × 10^−5^

^a^ AIC (Akaike’s information criterion) = 99.19. ^b^ *p* values < 0.05 indicate significance.

## Data Availability

The data presented in this study are available on request from the corresponding author.

## Supplementary Materials

The following are available online at https://www.mdpi.com/article/10.3390/life11111265/s1, Figure S1: Phylogenetic tree of *Rhizobium vitis* strains based on the neighbor-joining (NJ) method using the concatenated sequence data for *pyrG*, *recA*, and *rpoD*, Figure S2: Phylogenetic tree of *Allorhizobium* *vitis* strains based on the minimum-evolution (ME) method using the concatenated sequence data for *pyrG*, *recA*, and *rpoD*. Bootstrap values from 1000 samplings are indicated, Table S1: List of each category and sequence information.
