# Supplementary material for: Origin of Pathogens of Grapevine Crown Gall Disease in Hokkaido in Japan as Characterized by Molecular Epidemiology of Allorhizobium vitis Strains
Source: Life (Basel). 2021 Nov 19;11(11):1265. doi: 10.3390/life11111265 (PMC8620909; doi:10.3390/life11111265)
Supplement: Supplementary file 1 [file life-11-01265-s001.zip › life-1467466-supplementary-done.pdf]

Table S1. List of each category and sequence information

Strains (former name)	Ccu[c]	Cl[c]	Cp[c]	Cco[c]	Cy[c]	Cg[c]	Sequence data	Accession No.		
								pyrG	recA	rpoD
MAFF663001 (G-Ag-27)	1	7	6	1	1	5	Kawaguchi et al. 2008b	AB272143	AB272133	AB272151
MAFF212292 (YGAt32-3)	12	8	5	1	1	5	Kawaguchi et al. 2008b	AB272146	AB272138	AB272154
MAFF663017 (G-Ag-4)	1	9	8	1	1	5	Kawaguchi 2011	AB543812	AB543838	AB543865
MAFF663004 (G-Ag-9)	1	12	8	1	1	5	Kawaguchi 2011	AB543831	AB543858	AB543884
MAFF211676 (VAT03-9)	9	13	7	1	2	5	Kawaguchi et al. 2008b	AB272142	AB272131	AB272150
MAFF211944 (G-Ag-62)	1	15	4	1	1	5	Kawaguchi 2011	AB543815	AB543842	AB543868
MAFF211889 (G-Ag-52)	1	11	3	1	1	5	Kawaguchi et al. 2008b	AB272148	AB272135	AB272157
At-90-23	1	9	8	1	1	5	Kawaguchi 2011	AB543828	AB543855	AB543881
FZ-3-1	2	6	1	1	2	5	Kawaguchi 2011	AB543830	AB543857	AB543883
VAT20-30	5	3	1	1	4	5	This study	LC634605	LC634606	LC634607
VAT21-9	2	2	1	1	4	5	This study	LC635318	LC635319	LC635320
VAT21-10	3	2	1	1	4	5	This study	LC635321	LC635322	LC635323
VAT21-14	19	2	1	1	4	5	This study	LC635333	LC635334	LC635335
VAT21-15	19	2	1	1	4	5	This study	LC635336	LC635337	LC635338
ACME15	4	25	10	2	3	5	This study	LC629040	LC629041	LC629042
HNVR15	13	26	10	2	3	5	This study	LC629052	LC629053	LC629054
MAFF211909 (FM-3-2)	6	6	1	1	2	5	Kawaguchi 2011	AB543829	AB543856	AB543882
MAFF211913 (UM-1)	6	1	1	1	2	5	Kawaguchi 2011	AB543835	AB543862	AB543888
MAFF211301 (At-5)	1	9	8	1	1	6	Kawaguchi et al. 2008b	AB272144	AB272132	AB272152
MAFF211674 (A5-1)	1	5	3	1	2	6	Kawaguchi et al. 2008b	AB272145	AB272134	AB272153
MAFF211675 (A5-2)	1	5	3	1	2	6	Kawaguchi 2011	AB303682	AB303679	AB303678
MAFF211677 (A5-4)	1	5	3	1	2	6	Kawaguchi 2011	AB543811	AB543837	AB543864
A5-7	1	5	3	1	2	6	Kawaguchi 2011	AB543827	AB543854	AB543880
MAFF211302 (A5-8)	1	5	3	1	2	6	Kawaguchi 2011	AB543822	AB543849	AB543875
VAT06-11	11	14	7	1	2	6	Kawaguchi 2011	AB543824	AB543851	AB543877
MAFF663006 (G-Ag-19)	14	17	6	1	1	6	Kawaguchi 2011	AB543813	AB543839	AB543866
MAFF663007 (G-Ag-21)	1	17	6	1	1	6	Kawaguchi 2011	AB543814	AB543841	AB543867
MAFF663008 (G-Ag-23)	1	17	6	1	1	6	Kawaguchi 2011	AB543821	AB543848	AB543874
9-1-5	1	11	3	1	2	6	Kawaguchi et al. 2008b	AB272149	AB272136	AB272156
9-3-1	1	11	3	1	2	6	Kawaguchi 2011	AB543825	AB543852	AB543878
9-3-5	1	11	3	1	2	6	Kawaguchi 2011	AB543826	AB543853	AB543879
MAFF211943 (G-Ag-61)	15	18	2	1	1	6	Kawaguchi 2011	AB543832	AB543859	AB543885
MAFF211949 (G-Ag-67)	1	5	3	1	1	6	Kawaguchi 2011	AB543833	AB543860	AB543886
MAFF211910 (ISP-2)	5	19	1	1	2	6	This study	LC633188	LC633189	LC633190
MAFF212306 (VAR03-1)	9	10	7	1	2	2	Kawaguchi et al. 2008b	AB272139	AB272128	AB272158
ARK-1	10	10	7	1	2	2	Kawaguchi 2011	AB608979	AB608983	AB608986
MAFF212307 (VAR03-3)	9	10	7	1	2	2	Kawaguchi et al. 2008b	AB272140	AB272129	AB272159
MAFF212308 (VAR03-4)	9	10	7	1	2	2	Kawaguchi 2011	AB543823	AB543850	AB543876
ARK-2	10	10	7	1	2	2	Kawaguchi 2011	AB608980	AB608984	AB608985
ARK-3	10	10	7	1	2	2	Kawaguchi 2011	AB608981	AB608982	AB608987
MAFF212313 (VAR7-1)	9	10	7	1	2	2	Kawaguchi et al. 2008b	AB272141	AB272130	AB272160
VAR06-30	11	14	7	1	2	3	Kawaguchi 2011	AB285082	AB285087	AB285084
VAR06-31	11	14	7	1	2	3	Kawaguchi 2011	AB285083	AB285086	AB285085
DCCS15B	8	23	10	2	3	3	This study	LC629055	LC629056	LC629057
NCPPB3554[T]	18	29	13	3	1	1	Kawaguchi et al. 2008b	AB253130	AB253194	AB253258
DCCS15	8	23	10	2	3	1	This study	LC629046	LC629047	LC629048
MAFF211942 (G-Ag-60)	19	20	2	1	2	1	Kawaguchi et al. 2008b	AB272147	AB272137	AB272155
MAFF211918 (YHsM-2)	6	2	1	1	2	1	This study	LC633194	LC633195	LC633196
VAT07-1	11	13	7	1	2	1	Kawaguchi 2011	AB543817	AB543844	AB543870
NCPPB2562	17	30	12	4	1	1	Kawaguchi et al. 2008b	AB303681	AB303680	AB303677
MAFF211919 (YMK-1)	3	2	1	1	2	1	Kawaguchi 2011	AB543836	AB543863	AB543889
MAFF211920 (NKZ-2)	2	21	1	1	2	1	Kawaguchi 2011	AB543834	AB543861	AB543887
MAFF211908 (FK-2-2)	3	6	1	1	2	1	This study	LC633185	LC633186	LC633187
MAFF211915 (MM-2)	6	22	1	1	2	1	This study	LC633191	LC633192	LC633193
LCCH15	7	27	10	2	3	1	This study	LC629049	LC629050	LC629051
VAT20-1	2	1	1	1	4	1	This study	LC629123	LC629124	LC629125
VAT20-2	2	1	1	1	4	1	This study	LC633149	LC633150	LC633151
VAT20-3	3	1	1	1	4	1	This study	LC629126	LC629127	LC629128
VAT21-4	3	1	1	1	4	1	This study	LC633152	LC633153	LC633154
VAT21-5	3	1	1	1	4	1	This study	LC633155	LC633156	LC633157
VAT20-11	2	2	1	1	4	1	This study	LC629138	LC629139	LC629140
VAT20-12	3	2	1	1	4	1	This study	LC629141	LC629142	LC629143
VAT20-13	2	2	1	1	4	1	This study	LC629144	LC629145	LC629146
VAT20-21	3	4	1	1	4	1	This study	LC629147	LC629148	LC629149
VAT20-22	3	4	1	1	4	1	This study	LC629150	LC629151	LC629152
VAT20-23	3	4	1	1	4	1	This study	LC633158	LC633159	LC633160
VAT20-24	3	4	1	1	4	1	This study	LC633161	LC633162	LC633163
VAT20-25	3	4	1	1	4	1	This study	LC633164	LC633165	LC633166
VAT20-26	3	4	1	1	4	1	This study	LC633167	LC633168	LC633169
VAT20-31	5	3	1	1	4	1	This study	LC634608	LC634609	LC634610
VAT20-32	5	3	1	1	4	1	This study	LC635306	LC635307	LC635308
VAT21-1	2	2	1	1	4	1	This study	LC633170	LC633171	LC633172
VAT21-2	2	2	1	1	4	1	This study	LC633173	LC633174	LC633175
VAT21-3	2	2	1	1	4	1	This study	LC633176	LC633177	LC633178
VAT21-4	2	2	1	1	4	1	This study	LC633179	LC633180	LC633181
VAT21-5	2	2	1	1	4	1	This study	LC633182	LC633173	LC633174
VAT21-6	2	2	1	1	4	1	This study	LC635309	LC635310	LC635311
VAT21-7	2	2	1	1	4	1	This study	LC635312	LC635313	LC635314
VAT21-8	2	2	1	1	4	1	This study	LC635315	LC635316	LC635317
VAT20-7	2	1	1	1	4	4	This study	LC629129	LC629130	LC629131
VAT20-9	2	1	1	1	4	4	This study	LC629135	LC629136	LC629137
VAT21-11	2	1	1	1	4	4	This study	LC635324	LC635325	LC635326
VAT21-12	2	1	1	1	4	4	This study	LC635327	LC635328	LC635329
VAT21-13	3	1	1	1	4	4	This study	LC635330	LC635331	LC635332
VAT20-8	2	1	1	1	4	7	This study	LC629132	LC629133	LC629134
ZEME15	4	24	10	2	3	7	This study	LC629043	LC629044	LC629045
MAFF211912 (IS552-1)	5	19	1	1	2	7	Kawaguchi 2011	AB543820	AB543847	AB543873
MAFF211914 (UK-2)	3	1	1	1	2	7	Kawaguchi 2011	AB543818	AB543845	AB543871
NCPPB1771	16	28	11	5	1	7	Kawaguchi 2011	AB543816	AB543843	AB543869

[c] Ccu; Categorical number of cultivar, Cl; Categorical number of location of vineyard, Cp; Categorical number of prefecture or state, Cco; Categorical number of country, Cy; Categorical number of isolated year, Cg; Categorical number of genetic group.

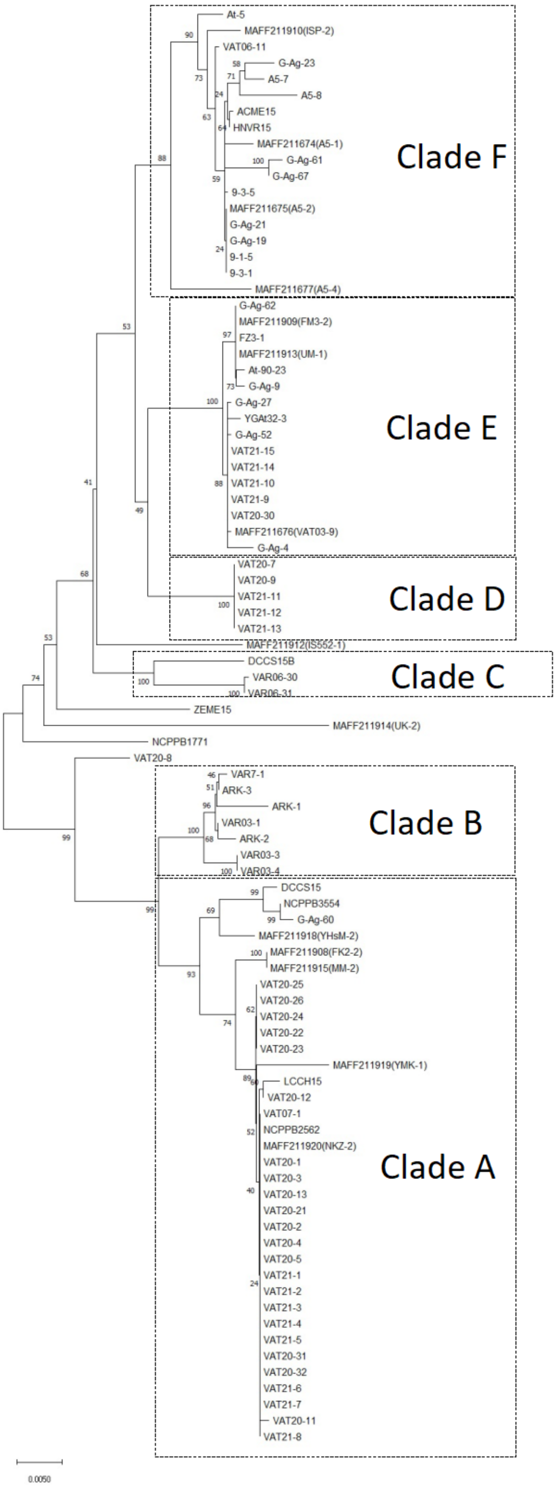
At-5
MAFF211910(ISP-2)
VAT06-11
G-Ag-23
A5-7
A5-8
ACME15
HNVR15
MAFF211674(A5-1)
G-Ag-61
G-Ag-67
9-3-5
MAFF211675(A5-2)
G-Ag-21
G-Ag-19
9-1-5
9-3-1
MAFF211677(A5-4)
Clade F
G-Ag-62
MAFF211909(FM3-2)
FZ3-1
MAFF211913(UM-1)
At-90-23
G-Ag-9
G-Ag-27
YGAt32-3
G-Ag-52
VAT21-15
VAT21-14
VAT21-10
VAT21-9
VAT20-30
MAFF211676(VAT03-9)
G-Ag-4
Clade E
VAT20-7
VAT20-9
VAT21-11
VAT21-12
VAT21-13
Clade D
MAFF211912(IS552-1)
DCCS15B
VAR06-30
VAR06-31
Clade C
ZEME15
MAFF211914(UK-2)
NCPPB1771
VAT20-8
VAR7-1
ARK-3
ARK-1
VAR03-1
ARK-2
VAR03-3
VAR03-4
Clade B
DCCS15
NCPPB3554
G-Ag-60
MAFF211918(YHsM-2)
MAFF211908(FK2-2)
MAFF211915(MM-2)
VAT20-25
VAT20-26
VAT20-24
VAT20-22
VAT20-23
MAFF211919(YMK-1)
LCCH15
VAT20-12
VAT07-1
NCPPB2562
MAFF211920(NKZ-2)
VAT20-1
VAT20-3
VAT20-13
VAT20-21
VAT20-2
VAT20-4
VAT20-5
VAT21-1
VAT21-2
VAT21-3
VAT21-4
VAT21-5
VAT20-31
VAT20-32
VAT21-6
VAT21-7
VAT20-11
VAT21-8
Clade A
0.0050

Figure S1

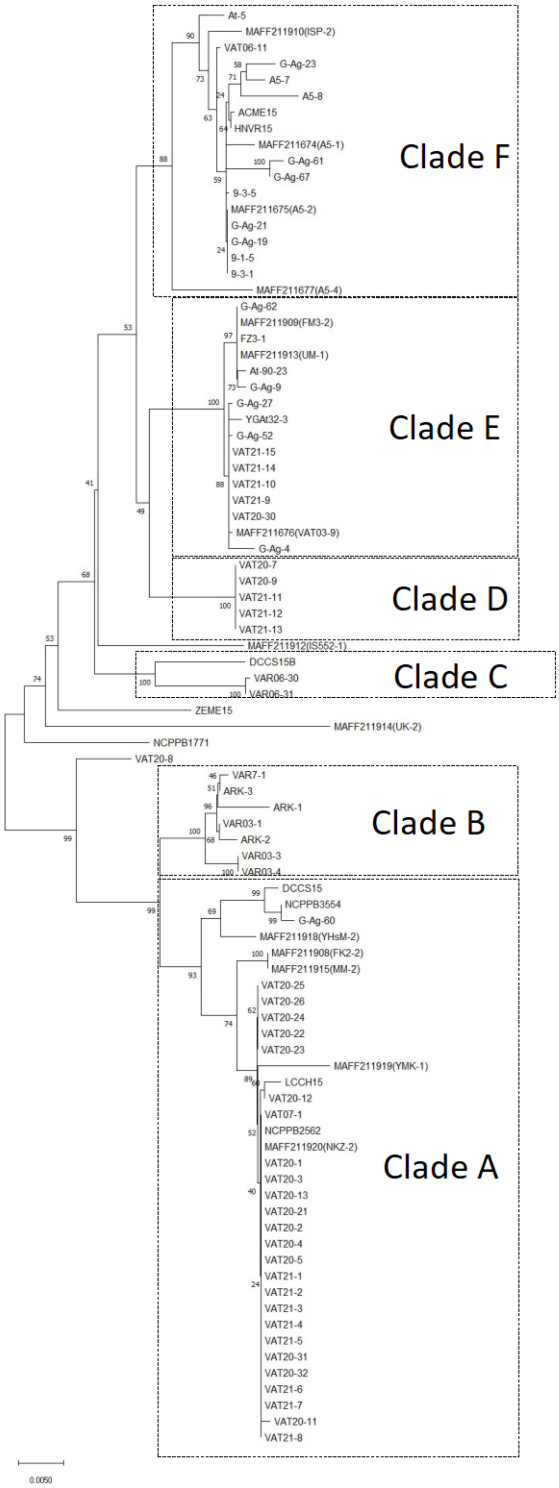
At-5
MAFF211910(ISP-2)
VAT06-11
G-Ag-23
A5-7
A5-8
ACME15
HNVR15
MAFF211674(A5-1)
G-Ag-61
G-Ag-67
9-3-5
MAFF211675(A5-2)
G-Ag-21
G-Ag-19
9-1-5
9-3-1
MAFF211677(A5-4)
Clade F
G-Ag-62
MAFF211909(FM3-2)
FZ3-1
MAFF211913(UM-1)
At-90-23
G-Ag-9
G-Ag-27
YGAt32-3
G-Ag-52
VAT21-15
VAT21-14
VAT21-10
VAT21-9
VAT20-30
MAFF211676(VAT03-9)
G-Ag-4
Clade E
VAT20-7
VAT20-9
VAT21-11
VAT21-12
VAT21-13
Clade D
MAFF211912(IS552-1)
DCCS15B
VAR06-30
VAR06-31
Clade C
ZEME15
MAFF211914(UK-2)
NCPPB1771
VAT20-8
VAR7-1
ARK-3
ARK-1
VAR03-1
ARK-2
VAR03-3
VAR03-4
Clade B
DCCS15
NCPPB3554
G-Ag-60
MAFF211918(YHsM-2)
MAFF211908(FK2-2)
MAFF211915(MM-2)
VAT20-25
VAT20-26
VAT20-24
VAT20-22
VAT20-23
MAFF211919(YMK-1)
LCCH15
VAT20-12
VAT07-1
NCPPB2562
MAFF211920(NKZ-2)
VAT20-1
VAT20-3
VAT20-13
VAT20-21
VAT20-2
VAT20-4
VAT20-5
VAT21-1
VAT21-2
VAT21-3
VAT21-4
VAT21-5
VAT20-31
VAT20-32
VAT21-6
VAT21-7
VAT20-11
VAT21-8
Clade A
0.0050

Figure S2
